# Trends, Characteristics, and Maternal Morbidity Associated With Unhoused Status in Pregnancy

**DOI:** 10.1001/jamanetworkopen.2023.26352

**Published:** 2023-07-31

**Authors:** Jessica M. Green, Sonya P. Fabricant, Christina J. Duval, Viraj R. Panchal, Sigita S. Cahoon, Rachel S. Mandelbaum, Joseph G. Ouzounian, Jason D. Wright, Koji Matsuo

**Affiliations:** 1 Division of Gynecologic Oncology, Department of Obstetrics and Gynecology, University of Southern California, Los Angeles; 2 Division of Obstetrics, Gynecology and Gynecologic Subspecialties, Department of Obstetrics and Gynecology, University of Southern California, Los Angeles; 3 Division of Reproductive Endocrinology and Infertility, Department of Obstetrics and Gynecology, University of Southern California, Los Angeles; 4 Division of Maternal-Fetal Medicine, Department of Obstetrics and Gynecology, University of Southern California, Los Angeles; 5 Division of Gynecologic Oncology, Department of Obstetrics and Gynecology, Columbia University College of Physicians and Surgeons, New York, New York; 6 Norris Comprehensive Cancer Center, University of Southern California, Los Angeles

## Abstract

**Importance:**

Unhoused status is a substantial problem in the US. Pregnancy characteristics and maternal outcomes of individuals experiencing homelessness are currently under active investigation to optimize health outcomes for this population.

**Objective:**

To assess the trends, characteristics, and maternal outcomes associated with unhoused status in pregnancy.

**Design, Setting, and Participants:**

This cross-sectional study analyzed data from the Healthcare Cost and Utilization Project National (Nationwide) Inpatient Sample. The study population included hospitalizations for vaginal and cesarean deliveries from January 1, 2016, to December 31, 2020. Unhoused status of these patients was identified from use of *International Statistical Classification of Diseases, Tenth Revision, Clinical Modification* code Z59.0. Statistical analysis was conducted from December 2022 to June 2023.

**Main Outcomes and Measures:**

Primary outcomes were (1) temporal trends; (2) patient and pregnancy characteristics associated with unhoused status, which were assessed with a multivariable logistic regression model; (3) delivery outcomes, including severe maternal morbidity (SMM) and mortality at delivery, which used the Centers for Disease Control and Prevention definition for SMM indicators and were assessed with a propensity score–adjusted model; and (4) choice of long-acting reversible contraception method and surgical sterilization at delivery.

**Results:**

A total of 18 076 440 hospital deliveries were included, of which 18 970 involved pregnant patients who were experiencing homelessness at the time of delivery, for a prevalence rate of 104.9 per 100 000 hospital deliveries. These patients had a median (IQR) age of 29 (25-33) years. The prevalence of unhoused patients increased by 72.1% over a 5-year period from 76.1 in 2016 to 131.0 in 2020 per 100 000 deliveries (*P* for trend < .001). This association remained independent in multivariable analysis. In addition, (1) substance use disorder (tobacco, illicit drugs, and alcohol use disorder), (2) mental health conditions (schizophrenia, bipolar, depressive, and anxiety disorders, including suicidal ideation and past suicide attempt), (3) infectious diseases (hepatitis, gonorrhea, syphilis, herpes, and COVID-19), (4) patient characteristics (Black and Native American race and ethnicity, younger and older age, low or unknown household income, obesity, pregestational hypertension, pregestational diabetes, and asthma), and (5) pregnancy characteristics (prior uterine scar, excess weight gain during pregnancy, and preeclampsia) were associated with unhoused status in pregnancy. Unhoused status was associated with extreme preterm delivery (<28-week gestation: 34.3 vs 10.8 per 1000 deliveries; adjusted odds ratio [AOR], 2.76 [95% CI, 2.55-2.99]); SMM at in-hospital delivery (any morbidity: 53.8 vs 17.7 per 1000 deliveries; AOR, 2.30 [95% CI, 2.15-2.45]); and in-hospital mortality (0.8 vs <0.1 per 1000 deliveries; AOR, 10.17 [95% CI, 6.10-16.94]), including case fatality risk after SMM (1.5% vs 0.3%; AOR, 4.46 [95% CI, 2.67-7.45]). Individual morbidity indicators associated with unhoused status included cardiac arrest (AOR, 12.43; 95% CI, 8.66-17.85), cardiac rhythm conversion (AOR, 6.62; 95% CI, 3.98-11.01), ventilation (AOR, 6.24; 95% CI, 5.03-7.74), and sepsis (AOR, 5.37; 95% CI, 4.53-6.36).

**Conclusions and Relevance:**

Results of this national cross-sectional study suggest that unhoused status in pregnancy gradually increased in the US during the 5-year study period and that pregnant patients with unhoused status were a high-risk pregnancy group.

## Key Points

**Question:**

What are the trends, characteristics, and maternal morbidity associated with unhoused status in pregnancy?

**Findings:**

In this cross-sectional study of 18 076 440 hospital deliveries, the prevalence rate of unhoused pregnant patients gradually increased by 72.1% from 2016 to 2020. Unhoused status in pregnancy was associated with comorbidity, substance use disorder, mental health conditions, infectious disease, and severe maternal morbidity and mortality at delivery.

**Meaning:**

Findings of this study suggest that pregnant patients with unhoused status are increasing, and these patients are a high-risk pregnancy group.

## Teaser

This cross-sectional study assesses patient and pregnancy factors as well as delivery outcomes for individuals experiencing homelessness during pregnancy.

## Introduction

Unhoused status, defined as lack of a fixed, regular, and adequate nighttime residence, is a complex problem affecting pregnant people in the US and has been associated with poor maternal and neonatal outcomes. Available data suggest that pregnant people with unhoused status are less likely than their counterparts with housed status to receive recommended prenatal and postnatal care^1,2,3^ and are more likely to access emergency health services and be hospitalized during pregnancy.^4,5^ Prior studies have reported that homelessness in pregnancy is associated with an increased risk of preterm birth,^2,4,6,7,8,9^ low birth weight,^1,2,7,8,9,10^ and neonatal intensive care unit admission.^1,2,7,10^ Limited data also suggest that pregnant patients with unhoused status are at an increased risk for chronic and infectious diseases,^3,10^ mental illness,^3^ substance use disorders,^1,3,8^ and intimate partner violence.^1^

Severe maternal morbidity (SMM) refers to unexpected, serious life-threatening medical conditions that can result in acute or chronic maternal injury or even fatal outcome in the perinatal period.^11^ Definitions of SMM vary and can include obstetric hemorrhage, organ failure, cardiovascular collapse, unplanned hysterectomy, eclampsia, and intensive care unit admission.^12^ In the US, the number of pregnant patients experiencing SMM is increasing steadily.^11^ While the exact explanation for this increase is unknown, increasing numbers of cesarean delivery, older maternal age, and maternal obesity are possible etiologies.^11^

Data remain limited regarding the association between unhoused status in pregnancy and SMM. Such data will guide future research to optimize health outcomes among pregnant people with unhoused status. The objective of this study was to assess the trends, characteristics, and maternal outcomes associated with unhoused status in pregnancy. 

## Methods

The University of Southern California Institutional Review Board deemed this cross-sectional study exempt from ethics review and the informed consent requirement because it used publicly available, deidentified data and involved no human research and public participation. We followed the Strengthening the Reporting of Observational Studies in Epidemiology (STROBE) reporting guideline.

### Data Source and Inclusion and Exclusion Criteria

We retrospectively queried the National (Nationwide) Inpatient Sample (NIS) of the Healthcare Cost and Utilization Project,^13^ which is supported and distributed by the Agency for Healthcare Research and Quality. The NIS data-capturing mechanism randomly samples approximately 20% of hospitalized patients every year from nearly 4500 participating hospitals across 48 states and the District of Columbia. The NIS captures a maximum of 40 diagnoses and 25 procedures for the index admission in each encounter. By applying the survey weights, the NIS represents more than 97% of hospital discharge data in the US population.

The study population included hospital deliveries (vaginal and cesarean) from January 1, 2016, to December 31, 2020. The starting point was chosen due to the introduction of the *International Statistical Classification of Diseases, Tenth Revision* (*ICD-10*) codes in the NIS. Patients aged 12 to 55 years were included. Unhoused status was identified from the use of *ICD-10 Clinical Modification* (*ICD-10-CM*) code Z59.0 (eTable 1 in Supplement 1). This study followed prior analyses that used the same coding schema,^14,15^ as recommended by the National Health Care for the Homeless Council.^16^ The code was unchanged in the *ICD-10-CM* schema throughout the study period.

### Main Outcomes

The following 4 outcomes were evaluated: (1) temporal trends; (2) patient and pregnancy characteristics; (3) delivery outcomes, including SMM and mortality at delivery; and (4) patient choice of long-acting reversible contraception (LARC) method and surgical sterilization at delivery. We adopted the Centers for Disease Control and Prevention definition for identifying the SMM indicators,^17^ which include a total of 21 diagnostic and procedural codes: acute myocardial infarction, acute kidney failure, adult respiratory distress syndrome, air and thrombotic embolism, amniotic fluid embolism, aneurysm, blood products transfusion, cardiac arrest or ventricular fibrillation, cardiac rhythm conversion, disseminated intravascular coagulation, eclampsia, heart failure or arrest during surgery or procedure, hysterectomy, puerperal cerebrovascular disorders, pulmonary edema or acute heart failure, severe anesthesia complications, sepsis, shock, sickle cell disease with crisis, temporary tracheostomy, and ventilation.

Delivery outcomes included gestational age at delivery and delivery route (vaginal or cesarean). The NIS captures mortality events occurring during the index hospital admission. Identification of LARC followed the instructions and prior investigations of the American College of Obstetricians and Gynecologists.^18,19,20,21^

### Study Covariates

The study covariates were preselected according to their relevance to the study objectives. These 51 covariates included 11 patient characteristics, 6 mental health conditions, 3 substance use disorders, 6 infectious diseases, 3 hospital parameters, and 22 pregnancy characteristics. Identification of these covariates was based on the Diagnosis-Related Group, *ICD-10-CM,* and *ICD-10 Procedure Coding System* codes (eTable 1 in Supplement 1). The covariates were grouped similarly in prior studies.^21,22,23,24^

Patient characteristics included (1) patient demographics (age, year, race and ethnicity, primary payer, and census-level median household income), (2) medical comorbidities (obesity, pregestational hypertension, pregestational diabetes, and asthma), and (3) gynecological factors (uterine myoma and uterine anomaly). Race and ethnicity categories (Asian or Pacific Islander, Black, Hispanic, Native American, White, and others) were determined by the NIS and were examined because of their association with pregnancy and delivery characteristics and outcomes.

Mental health conditions included schizophrenia disorder, bipolar disorder, depressive disorder, anxiety disorder, adjustment disorder, and suicidal ideation (including past suicide attempt). Substance use disorders included tobacco use disorder, illicit drug use disorder, and alcohol use disorder. Infectious diseases included gonorrhea, syphilis, hepatitis virus, anogenital herpes, tuberculosis, and COVID-19.^25^ Hospital parameters included relative bed capacity, hospital location and teaching status, and region. Pregnancy characteristics included fetal factors (multifetal gestation, breech presentation, large for gestational age, intrauterine growth restriction, intrauterine fetal demise, fetal anomaly, polyhydramnios, and oligohydramnios), membranous factors (preterm premature rupture of membrane and intrauterine infection), umbilical cord factor (umbilical cord prolapse), placental factors (placental abruption, placenta previa, and placenta accreta spectrum), uterine factors (prior uterine scar and uterine rupture), past obstetric history (grand multiparity and prior pregnancy loss), and maternal factors (gestational hypertension, preeclampsia, gestational diabetes, and excess maternal weight gain during pregnancy).

### Statistical Analysis

The first step of analysis was to assess the temporal trends of unhoused status in pregnancy from January 1, 2016, to December 31, 2020. Cases were aggregated per calendar year, and the Cochran-Armitage test was used to examine the trend in the annual prevalence rate of pregnancy among patients with unhoused status.

The second step of analysis was to identify the independent patient, pregnancy, and hospital characteristics associated with unhoused status in pregnancy. A binary logistic regression model was fitted for multivariable analysis. Covariates with *P* < .05 in univariable analysis were considered in the modeling. Multicollinearity was assessed among the study covariates. The effect size for unhoused status in pregnancy was expressed with an adjusted odds ratio (AOR) and a corresponding 95% CI.

The third step of analysis was to evaluate delivery outcomes associated with unhoused status in pregnancy. Outcome event rates were reported per 1000 deliveries. The risk estimates for unhoused pregnancy were adjusted for propensity score.^26^ A binary logistic regression model was used to compute the propensity score, and the study covariates that were associated with unhoused status in pregnancy and historically known for SMM were entered in the modeling.^11^ The effect size on outcome measures for the unhoused group compared with the housed group was expressed with an AOR and a corresponding 95% CI.

Various sensitivity analyses were undertaken to assess the robustness of the study findings. First, we examined trends and outcomes in subcohorts that were relevant to the study objective, including age, region, comorbidity, mental health condition, and substance use disorder (a total of 25 subcohorts). Second, we assessed SMM for individual morbidity indicators excluding blood product transfusion and hysterectomy, similar to the process in prior studies, including the Centers for Disease Control and Prevention analysis.^11^ Third, we examined (1) case fatality rate, defined as the mortality among patients with SMM^27^; (2) selected high-risk mortality, defined as more than 3-fold odds for morbidities in the unhoused group; and (3) prolonged hospital stay, defined as the length of admission for delivery of 7 days or more.

Statistical analysis was conducted from December 2022 to June 2023. The weights for national estimates provided by the NIS were used for analysis. Statistical interpretation followed a 2-tailed hypothesis, and a 2-sided *P* < .05 was considered to be statistically significant. Cases with unknown data were grouped as 1 category in each variable. IBM SPSS Statistics, version 28.0 (IBM Corp), and R, version 3.5.3 (R Foundation for Statistical Computing) were used for all analysis.

## Results

A total of 18 076 440 hospital deliveries for national estimates were examined. Patients had a median (IQR) age of 29 (25-33) years and included individuals with Asian or Pacific Islander (5.9%), Black (14.5%), Hispanic (20.0%), Native American (0.7%), White (50.4%), and other or unknown (8.5%) race and ethnicity. Most patients were privately insured (51.5%). Most deliveries had full-term gestation (88.6%), were vaginal birth (67.7%), and occurred at an urban teaching center (69.9%).

Patients with unhoused status accounted for 18 970 of the deliveries, corresponding with a prevalence rate of 104.9 per 100 000 deliveries. In other words, 1 in 952 hospital deliveries were for pregnant patients with unhoused status.

### Temporal Trends

The prevalence of unhoused pregnant people increased by 72.1% over a 5-year period from 76.1 in 2016 to 131.0 per 100 000 deliveries in 2020 (*P* for trend < .001) (Table 1). This result corresponds with an increase in the prevalence of unhoused status in pregnancy from 1 in 1314 deliveries in 2016 to 1 in 764 deliveries in 2020.

**Table 1.** Trends Associated With Unhoused Status in Pregnancy From 2016 to 2020

Characteristic	Annual prevalence per 100 000 deliveries					Interval increase, %^a^	*P* value^b^
	2016	2017	2018	2019	2020		
Whole cohort	76.1	88.4	113.9	118.4	131.0	72.1	<.001
Age, y							
<25	93.3	109.9	130.0	142.0	156.6	67.8	<.001
25-29	76.1	83.5	121.8	123.4	141.8	86.3	<.001
30-34	63.6	80.2	90.9	99.9	111.6	75.5	<.001
≥35	70.5	79.3	116.0	109.7	114.4	62.3	<.001
Race and ethnicity^c^							
Asian or Pacific Islander	31.8	51.3	27.5	42.0	40.2	26.4	.60
Black	167.6	189.0	277.1	246.6	281.5	68.0	<.001
Hispanic	53.4	70.0	83.2	93.1	113.1	111.8	<.001
Native American	130.5	273.3	516.4	452.0	527.8	304.4	<.001
White	63.5	66.9	90.2	95.2	100.9	58.9	<.001
Region							
Northeast	70.0	92.6	132.4	111.8	164.1	134.4	<.001
Midwest	64.0	72.2	97.9	122.4	118.0	84.4	<.001
South	47.9	54.2	68.2	69.2	73.6	53.7	<.001
West	136.0	155.0	192.1	202.0	218.0	60.3	<.001
Medical comorbidity							
Obesity	133.2	145.8	155.8	154.7	162.4	21.9	<.001
Asthma	273.2	317.3	378.1	341.9	334.7	22.5	<.001
Pregestational hypertension	161.5	266.5	244.3	222.5	198.9	23.2	<.001
Mental health condition							
Any	476.5	459.4	597.2	560.4	547.9	15.0	<.001
Schizophrenia disorder	5427.4	5858.9	6025.5	5056.2	5843.4	7.7	.99
Bipolar disorder	1227.0	1247.2	1758.0	1714.5	1823.7	48.6	<.001
Depressive disorder	351.7	388.2	523.2	462.7	442.6	25.8	<.001
Anxiety disorder	326.5	296.3	353.8	386.8	345.6	5.8	.01
Adjustment disorder	866.3	904.0	1177.6	1201.5	1341.7	54.9	.01
Suicidal ideation and attempt	2626.3	2926.2	3106.2	3035.0	2994.4	14.0	.48
Substance use disorder							
Any	671.3	801.2	1034.0	1045.9	1203.3	79.2	<.001
Tobacco use disorder	609.7	727.9	951.7	929.1	1156.5	89.7	<.001
Alcohol use disorder	2500.0	2814.1	3205.1	2626.8	3886.3	55.5	<.001
Illicit drug use	1357.4	1547.7	1924.1	1933.9	2149.2	58.3	<.001
Any mental health condition or substance use disorder	495.0	536.1	657.8	638.2	645.4	30.4	<.001

^a^ Proportional change from 2016 to 2020.

^b^ *P* values were calculated using Cochran-Armitage trend test.

^c^ Race and ethnicity data were obtained from the National (Nationwide) Inpatient Sample. Data for other (designated by the National Inpatient Sample) or unknown race and ethnicity was not clinically relevant.

By age group, patients aged 25 to 29 years had the largest interval increase in unhoused status vs those in other age groups (86.3% vs 62.3%-75.5%). By race and ethnicity, Native American patients had the largest interval increase among all racial and ethnic groups (304.4% vs 26.4%-111.8%). By region, residents of the Northeast had the largest interval increase compared with residents in other regions (134.4% vs 53.7%-84.4%) (Table 1).

### Patient Characteristics

The increasing prevalence over time of unhoused status at delivery remained robust in a multivariable analysis (Table 2). The AORs were 1.09 (95% CI, 1.03-1.15) for 2017, 1.32 (95% CI, 1.26-1.39) for 2018, 1.33 (95% CI, 1.27-1.40) for 2019, and 1.44 (95% CI, 1.37-1.51) for 2020 compared with 2016.

**Table 2.** Patient Characteristics Associated With Unhoused Status in Pregnancy

Characteristic	Patients, No. (%)	Prevalence per 100 000 deliveries	AOR (95% CI)^a^
All hospital deliveries	18 076 440 (100)	104.9	
Age, y			
<25	4 395 508 (24.3)	124.7	1.06 (1.02-1.11)
25-29	5 211 237 (28.8)	108.3	0.98 (0.94-1.02)
30-34	5 193 037 (28.7)	89.1	1 [Reference]
≥35	3 276 659 (18.1)	98.3	1.08 (1.03-1.13)
Year			
2016	3 756 851 (20.8)	76.1	1 [Reference]
2017	3 699 411 (20.5)	88.4	1.09 (1.03-1.15)
2018	3 627 299 (20.1)	113.9	1.32 (1.26-1.39)
2019	3 564 109 (19.7)	118.4	1.33 (1.27-1.40)
2020	3 428 770 (19.0)	131.0	1.44 (1.37-1.51)
Race and ethnicity^b^			
Asian or Pacific Islander	1 075 534 (5.9)	38.6	0.98 (0.88-1.08)
Black	2 614 975 (14.5)	231.7	2.68 (2.55-2.82)
Hispanic	3 619 174 (20.0)	82.3	1 [Reference]
Native American	127 685 (0.7)	375.9	1.30 (1.17-1.44)
White	9 110 779 (50.4)	83.0	1.25 (1.19-1.31)
Other^c^	793 730 (4.4)	82.5	1.38 (1.27-1.51)
Unknown	734 564 (4.1)	111.0	1.88 (1.73-2.04)
Primary payer			
Medicaid	76 59 051 (42.4)	206.0	1.08 (1.01-1.15)
Private	9 309 720 (51.5)	13.2	0.15 (0.14-0.17)
Self-pay	457 210 (2.5)	188.1	1.16 (1.06-1.28)
Other	629 295 (3.5)	171.6	1 [Reference]
Unknown	21 165 (0.1)	141.7	1.21 (0.84-1.75)
Household income			
Quartile 1 (lowest)	5 008 078 (27.7)	135.3	1.16 (1.10-1.23)
Quartile 2	4 550 222 (25.2)	88.1	1.06 (1.00-1.13)
Quartile 3	4 420 557 (24.5)	70.6	1.04 (0.98-1.10)
Quartile 4 (highest)	3 935 093 (21.8)	42.3	1 [Reference]
Unknown	162 490 (0.9)	2092.4	19.45 (18.24-20.74)
Obesity			
No	16 002 761 (88.5)	98.9	1 [Reference]
Yes	2 073 679 (11.5)	151.4	1.08 (1.03-1.12)
Asthma			
No	17 120 216 (94.7)	92.3	1 [Reference]
Yes	956 225 (5.3)	330.5	1.49 (1.43-1.55)
Tobacco use disorder			
No	17 133 626 (94.8)	63.3	1 [Reference]
Yes	942 814 (5.2)	861.8	3.12 (3.01-3.24)
Alcohol use disorder			
No	18 050 010 (>99.9)	100.7	1 [Reference]
Yes	26 430 (<0.1)	3007.9	2.01 (1.85-2.19)
Illicit drug use disorder			
No	17 582 520 (97.3)	57.3	1 [Reference]
Yes	493 920 (2.7)	1799.9	6.31 (6.08-6.55)
Schizophrenia disorder			
No	18 055 700 (>99.9)	98.6	1 [Reference]
Yes	20 740 (<0.1)	5641.3	4.95 (4.59-5.33)
Bipolar disorder			
No	17 927 580 (99.2)	92.7	1 [Reference]
Yes	148 860 (0.8)	1578.7	2.74 (2.59-2.89)
Depressive disorder			
No	17 415 920 (96.3)	92.2	1 [Reference]
Yes	660 520 (3.7)	439.8	1.92 (1.83-2.01)
Anxiety disorder			
No	17 236 286 (95.4)	93.2	1 [Reference]
Yes	840 155 (4.6)	345.8	1.16 (1.11-1.22)
Adjustment disorder			
No	18 051 880 (>99.9)	103.6	1 [Reference]
Yes	24 560 (<0.1)	1119.7	3.00 (2.62-3.44)
Suicidal ideation and attempt			
No	18 051 440 (>99.9)	101.0	1 [Reference]
Yes	25 000 (<0.1)	2980.0	3.03 (2.78-3.31)
Gonorrhea			
No	18 068 345 (>99.9)	103.6	1 [Reference]
Yes	8095 (<0.1)	3211.9	3.87 (3.34-4.48)
Syphilis			
No	18 062 320 (>99.9)	103.4	1 [Reference]
Yes	14 120 (<0.1)	2089.2	2.44 (2.12-2.81)
Hepatitis virus			
No	17 957 550 (99.3)	97.5	1 [Reference]
Yes	118 890 (0.7)	1236.4	1.56 (1.46-1.66)
Anogenital herpes			
No	17 820 185 (98.6)	103.0	1 [Reference]
Yes	256 255 (1.4)	241.9	1.20 (1.10-1.31)
COVID-19			
No	18 030 715 (99.7)	104.6	1 [Reference]
Yes	45 725 (0.3)	240.6	1.82 (1.49-2.22)
Grand multiparity			
No	18 019 130 (99.7)	104.4	1 [Reference]
Yes	57 310 (0.3)	261.7	1.28 (1.08-1.52)
Prior pregnancy losses			
No	18 024 695 (99.7)	104.8	1 [Reference]
Yes	51 745 (0.3)	154.6	1.06 (0.84-1.34)
Prior uterine scar			
No	14 818 972 (82.0)	99.7	1 [Reference]
Yes	3 257 468 (18.0)	128.8	1.09 (1.05-1.13)
Uterine myoma			
No	17 811 155 (98.5)	105.4	1 [Reference]
Yes	265 285 (1.5)	71.6	0.68 (0.58-0.79)
Uterine anomaly			
No	18 000 920 (99.6)	105.1	1 [Reference]
Yes	75 520 (0.4)	66.2	0.74 (0.56-0.98)

Abbreviation: AOR, adjusted odds ratio.

^a^ A binary logistic regression model was used for multivariable analysis. All covariates were entered in the model and all were statistically significant in univariable analysis.

^b^ Race and ethnicity data were obtained from the National (Nationwide) Inpatient Sample.

^c^ Other was designated by the National Inpatient Sample.

In addition, the following factors were associated with unhoused status in pregnancy: (1) substance use disorders (tobacco, illicit drugs, and alcohol); (2) mental health conditions (schizophrenia, bipolar, depressive, and anxiety disorders, including suicidal ideation and past attempt); (3) infectious diseases (hepatitis virus, gonorrhea, syphilis, anogenital herpes, and COVID-19); and (4) patient characteristics (younger and older age, Black and Native American race and ethnicity, low or unknown household income, obesity, pregestational hypertension, pregestational diabetes, and asthma); and (5) pregnancy characteristics (excess weight gain during pregnancy, gestational hypertension, prior uterine scar, and preeclampsia) (Table 2 and Table 3; eTable 2 in Supplement 1).

**Table 3.** Pregnancy Characteristics Associated With Unhoused Status

Characteristic	No. (%)	Prevalence per 100 000 deliveries	AOR (95% CI)^a^
All hospital deliveries	18 076 440 (100)	104.9	NA
Hypertension			
No	15 437 246 (85.4)	91.9	1 [Reference]
Pregestational	398 390 (2.2)	194.5	1.19 (1.10-1.29)
Gestational	997 324 (5.5)	136.9	1.24 (1.17-1.32)
Preeclampsia	1 137 165 (6.3)	207.1	1.42 (1.36-1.49)
NOS	106 315 (0.6)	268.1	1.56 (1.38-1.77)
Diabetes			
No	16 409 266 (90.8)	107.6	1 [Reference]
Pregestational	199 555 (1.1)	215.5	1.19 (1.07-1.32)
Gestational	1 444 029 (8.0)	57.8	0.67 (0.62-0.72)
NOS	23 590 (0.1)	212.0	1.36 (1.02-1.81)
Excess maternal weight gain			
No	18 022 725 (99.7)	104.8	1 [Reference]
Yes	53 715 (0.3)	167.6	1.39 (1.12-1.72)
Multifetal gestation			
No	1 7752 940 (98.2)	104.3	1 [Reference]
Yes	323 500 (1.8)	137.6	1.12 (1.02-1.24)
Breech presentation			
No	17 399 805 (96.3)	103.4	1 [Reference]
Yes	676 635 (3.7)	143.4	1.08 (1.01-1.16)
LGA			
No	17 588 440 (97.3)	106.4	1 [Reference]
Yes	488 000 (2.7)	53.3	0.82 (0.72-0.93)
IUGR			
No	17 427 360 (96.4)	101.3	1 [Reference]
Yes	649 080 (3.6)	202.6	1.03 (0.97-1.09)
Intrauterine fetal demise			
No	17 933 620 (99.2)	103.4	1 [Reference]
Yes	142 820 (0.8)	294.1	1.34 (1.21-1.49)
Fetal anomaly			
No	17 874 510 (98.9)	104.1	1 [Reference]
Yes	201 930 (1.1)	180.8	1.03 (0.92-1.15)
Polyhydramnios			
No	17 802 095 (98.5)	104.5	1 [Reference]
Yes	274 345 (1.5)	131.2	1.08 (0.97-1.21)
Oligohydramnios			
No	17 600 130 (97.4)	103.7	1 [Reference]
Yes	476 310 (2.6)	150.1	1.17 (1.08-1.27)
PPROM			
No	17 592 500 (97.3)	100.3	1 [Reference]
Yes	483 940 (2.7)	272.8	1.33 (1.25-1.41)
Intrauterine infection			
No	17 649 230 (97.6)	104.4	1 [Reference]
Yes	427 210 (2.4)	128.7	1.13 (1.04-1.24)
Placental abruption			
No	17 874 740 (98.9)	102.6	1 [Reference]
Yes	201 700 (1.1)	314.8	1.14 (1.05-1.25)
Placenta accreta spectrum			
No	18 053 855 (>99.9)	104.7	1 [Reference]
Yes	22 585 (<0.1)	309.9	1.48 (1.15-1.90)

Abbreviations: AOR, adjusted odds ratio; IUGR, intrauterine growth restriction; LGA, large for gestational age; NA, not applicable; NOS, not otherwise specified; PPROM, preterm premature rupture of membrane.

^a^ A binary logistic regression model was used for multivariable analysis. All covariates were entered in the model and all were statistically significant in univariable analysis.

### Maternal Outcomes

The maternal outcomes at delivery for the unhoused and housed groups included SMM (any morbidity: 53.8 vs 17.7 per 1000 deliveries; AOR, 2.30 [95% CI, 2.15-2.45]), mortality (0.8 vs <0.1 per 1000 deliveries; AOR, 10.17 [95% CI, 6.10-16.94]), and case fatality following SMM (1.5% vs 0.3%; AOR, 4.46 [95% CI, 2.67-7.45]) (Table 4). The association for SMM was consistent in several subgroups per patient characteristics (eTable 3 in Supplement 1).

**Table 4.** SMM and Mortality Associated With Unhoused Status in Pregnancy

	Outcome rates per 1000 deliveries		AOR (95% CI)^a^^,^^b^
	Housed group	Unhoused group	
Composite SMM factors			
Any^c^	17.7	53.8	2.30 (2.15-2.45)
Except for blood transfusion	7.7	30.0	2.79 (2.57-3.04)
Except for blood transfusion or hysterectomy	7.0	28.2	2.92 (2.68-3.19)
Core morbidity^d^	3.6	20.0	3.84 (3.45-4.27)
Individual morbidity indicator^e^			
Cardiac arrest or ventricular fibrillation	<0.1	1.6	12.43 (8.66-17.85)
Cardiac rhythm conversion	<0.1	0.8	6.62 (3.98-11.01)
Ventilation	0.5	4.5	6.24 (5.03-7.74)
Sepsis	1.1	7.1	5.37 (4.53-6.36)
Adult respiratory distress syndrome	0.9	6.1	4.52 (3.75-5.44)
Acute kidney failure	1.4	8.4	3.96 (3.38-4.65)
Air and thrombotic embolism	0.3	1.3	3.25 (2.20-4.83)
Pulmonary edema or acute heart failure	0.7	3.4	3.13 (2.45-4.01)
Shock	0.8	2.9	2.79 (2.14-3.65)
Hysterectomy	0.9	3.2	2.29 (1.78-2.96)
Eclampsia	1.4	3.7	2.08 (1.64-2.63)
Blood products transfusion	11.4	29.3	1.93 (1.79-2.13)
Coagulopathy	1.7	3.7	1.73 (1.37-2.20)
Other outcome measures			
Death^f^	<0.1	0.8	10.17 (6.10-16.94)
Case fatality rate, %^g^	0.3	1.5	4.46 (2.67-7.45)
Preterm delivery: <37 wk^h^	100.7	262.8	2.48 (2.40-2.57)
Early preterm delivery: <34 wk^h^	31.4	108.6	2.99 (2.85-3.13)
Extreme preterm delivery: <28 wk^h^	10.8	34.3	2.76 (2.55-2.99)
Cesarean delivery	323.8	337.4	1.06 (1.03-1.10)
Postpartum hemorrhage	40.0	64.0	1.43 (1.35-1.52)
Hospital stay ≥7 d	15.5	80.7	3.83 (3.63-4.05)

Abbreviations: AOR, adjusted odds ratio; SMM, severe maternal morbidity.

^a^ Adjusted for the propensity score generated by the independent characteristics associated with unhoused status that were also historically known as the risk factors for severe maternal morbidity: age, year, race and ethnicity, obesity, hypertension, diabetes, prior uterine scar, COVID-19, placental abruption, placental accreta spectrum, and hospital teaching status.

^b^ Effect size for unhoused vs housed status on outcome measures, with the housed group as the referent.

^c^ Included any 1 of the 21 indicators for SMM per the Centers for Disease Control and Prevention definition: acute myocardial infarction, aneurysm, acute kidney failure, adult respiratory distress syndrome, amniotic fluid embolism, cardiac arrest or ventricular fibrillation, cardiac rhythm conversion, disseminated intravascular coagulation, eclampsia, heart failure or arrest during surgery or procedure, puerperal cerebrovascular disorders, pulmonary edema or acute heart failure, severe anesthesia complications, sepsis, shock, sickle cell disease with crisis, air and thrombotic embolism, blood products transfusion, hysterectomy, temporary tracheostomy, or ventilation.

^d^ Any of the following indicators that had an AOR greater than 3.

^e^ Morbidity indicators are displayed in descending order for the unhoused group. Indicators with a small number of events are not listed.

^f^ During the admission for delivery.

^g^ In-hospital mortality rate among SMM.

^h^ Compared with 37 weeks’ gestation or later.

Individual morbidity indicators associated with unhoused status in pregnancy included cardiac arrest (AOR, 12.43; 95% CI, 8.66-17.85), cardiac rhythm conversion (AOR, 6.62; 95% CI, 3.98-11.01), ventilation (AOR, 6.24; 95% CI, 5.03-7.74), and sepsis (AOR, 5.37; 95% CI, 4.53-6.36) (Table 4). Other outcome measures associated with unhoused status in pregnancy included extreme preterm delivery (<28 weeks’ gestation: 34.3 vs 10.8 per 1000 deliveries; AOR, 2.76 [95% CI, 2.55-2.99]), postpartum hemorrhage (64.0 vs 40.0 per 1000 deliveries; AOR, 1.43 [95% CI, 1.35-1.52]), and prolonged hospital stay (≥7 days: 80.7 vs 15.5; AOR, 3.83 [95% CI, 3.63-4.05]).

### Contraceptive and Sterilization Choices

Among pregnant patients who did not have a hysterectomy, those in the unhoused group were more likely to choose LARC compared with those in the housed group (subdermal contraceptive implant: 32.1 vs 3.5 per 1000 deliveries; AOR, 6.91 [95% CI, 6.31-7.55]; intrauterine device: 30.9 vs 5.6 per 1000 deliveries; AOR, 4.20 [95% CI, 3.84-4.60]) (eTable 4 in Supplement 1).

## Discussion

Key results of the current study were as follows: (1) the prevalence of pregnant people with unhoused status in the US increased significantly over the study period from 2016 to 2020, and (2) unhoused status in pregnancy was associated with high-risk pregnancy characteristics. Several areas deserve further discussion.

During the 5-year study period, there was an overall 72.1% increase in the prevalence of pregnant people with unhoused status. The interval increase in unhoused status in pregnancy appeared to be higher compared with the general population in the US during the study period (5.5% for unhoused status, including 42% for chronic unhoused status^28^). Lack of affordable housing options, the deinstitutionalization of psychiatric care, and changes to legislation that previously diverted individuals through substance use disorder treatment programs are some of the proposed factors in this trend.^29,30^

Given the paucity of data on trends of unhoused status in pregnancy, the observed results from the current study add important information to the literature. While we observed an interval increase in the prevalence of pregnant patients with unhoused status from all racial and ethnic groups, Native American individuals experienced the greatest proportional interval increase over the study period, and the relative increase was far higher than for other groups. Native American individuals have been identified as having a disproportionate burden of unhoused status compared with other segments of the US population,^31^ and this experience appeared to also be present in the pregnant population.

Unhoused status in pregnancy was more likely to occur among individuals younger than 25 years and those 35 years or older. While a prior study in California found an increased prevalence among people younger than 18 years from 2007 to 2012,^4^ this 2016 to 2020 nationwide analysis identified an additional association with older maternal age (≥35 years). While the explanation for this result is unknown, this information warrants further investigation and calls for greater public awareness. Moreover, Black and Native American individuals experienced the highest prevalence of unhoused status in pregnancy. Systemic racism in housing availability as well as employment and wages likely play a role in this trend.^32^

Unhoused status in pregnancy was associated with substance use disorders and mental health disorders. Conversely, such factors were likely exacerbated by the chronic stress of homelessness.^29^ This finding is consistent with the existing literature^3,4,6^ and highlights the importance of improving access to psychiatric and substance use treatment and diversion services for pregnant people.

Additionally, unhoused status in pregnancy was associated with sexually transmitted infections. While the sexual and romantic decision-making of unhoused people is as varied as that for housed people,^33^ this trend may be explained by pregnant patients with unhoused status being at an increased risk of sexual violence and being more likely to have multiple sexual partners.^34,35^ Barriers to accessing treatment are another possible factor.

Unhoused status in pregnancy was associated with preterm delivery, including early preterm (<34 weeks) and extremely preterm (<28 weeks) deliveries. Administrative data did not permit us to differentiate medically indicated preterm delivery from spontaneous preterm birth, and the finding likely reflects a combination of these 2 etiologies.

Previous studies have reported an association between unhoused status in pregnancy and preterm birth (<37 weeks)^2,4,6,7,8,9^; however, few studies have examined preterm birth among subgroups with early gestational age. A study in California between 2007 and 2012 found that unhoused status was associated with preterm birth at 32 to 36 weeks (AOR, 1.3; 95% CI, 1.1-1.5) but not before 32 weeks (AOR, 1.0; 95% CI, 0.7-1.4).^4^ The difference between this past result and the current results may be attributed to differences in their study populations (singleton births only vs all delivery types), state and regional factors, or temporal factors (2007-2012 vs 2016-2020).

Overall, unhoused status was associated with SMM at hospital delivery. This finding is, in part, consistent with a recent study reporting the association between housing insecurity and SMM, including mortality during hospital admission for delivery.^6^ The current study, compared with the previous study,^6^ found an association between case fatality risk and SMM among patients with unhoused status at delivery. It may be possible that the increased rates of underlying medical comorbidities among unhoused pregnant patients were factors in the inability to resuscitate patients after severe complications. Additionally, race and ethnicity other than White race were associated with failure to rescue, independent of other patient factors.^27,36^ A disproportionate number of pregnant patients with unhoused status were from racial and ethnic minority groups, suggesting that systemic factors, such as practitioner bias, may play a role in the findings.

The core individual morbidity indicators associated with unhoused status in pregnancy were mainly cardiopulmonary complications. We were not able to elucidate the underlying mechanism of this association, but it is likely multifactorial.

Several factors may play a role in the maternal morbidity among pregnant people with unhoused status. They include increased rates of medical and psychiatric comorbidities^3,10^; decreased access to routine prenatal and postpartum care^1,2,3^; lack of social support structures^37^; and systemic bias from health care practitioners, hospitals, and health systems.^38^

Unhoused pregnant patients were more likely to choose LARC methods for postpartum contraception. A possible explanation for this result could be practitioner bias when providing contraception counseling.^39^ Although the prevalence of LARC use is comparatively lower than other contraceptive methods for the population as a whole,^40^ the higher use of LARC methods in the current study may highlight the wider national-practice shift in contraception counseling and provision toward LARC methods.^41^

### Limitations

Key limitations of this study included unmeasured bias with lack of information on the unhoused status (underlying reason, setting, and duration); cause of death; use of antenatal care, such as gestational medical screening, indication of delivery; neonatal information; medication use and type for mental health condition; and postdischarge data for the postpartum period. Causality of and chronological relationship to certain risk factors were unknown. Identification of unhoused pregnancy status was based solely on administrative coding, and the accuracy of data, including unhoused status (*ICD-10-CM* code Z59.0), was not assessable without medical record review. The observed increase in the prevalence of unhoused status in pregnancy may be associated with the increase in the use of *ICD-10-CM* code Z59.0, as recommended by the National Health Care for the Homeless Council in October 2016.^16^ Similarly, the accuracy of study covariates for behavioral factors, pregnancy characteristics, and medical comorbidities was not assessable and may have been underreported.

Other limitations included lack of information on individual-level socioeconomic status, nonhospital deliveries, multiple statistical comparisons, and possible multiple entries for the same pregnant patients. Ascertainment bias and unknown generalizability to other populations and countries were also limitations.

## Conclusions

This national, serial cross-sectional analysis found that unhoused status in pregnancy gradually increased in the US from 2016 to 2020 and that substantial maternal outcomes were associated with this phenomenon. These findings support safe housing for all pregnant people as an important factor in improving perinatal outcomes. Recognizing that pregnant individuals with unhoused status are a high-risk pregnancy group is necessary. Further studies are warranted to validate the results of the current study and to identify the causes of SMM among unhoused pregnant patients.

## Notes

Supplement 1. **eTable 1.** Coding Information **eTable 2.** Hospital Factors Associated With Unhoused Pregnancy **eTable 3.** Sensitivity Analysis for Severe Maternal Morbidity **eTable 4.** Contraceptive and Sterilization Choices Among Unhoused Pregnant Patients

Supplement 2.
Data Sharing Statement
