# Supplementary material for: Trends, Characteristics, and Maternal Morbidity Associated With Unhoused Status in Pregnancy
Source: JAMA Netw Open. 2023 Jul 31;6(7):e2326352. doi: 10.1001/jamanetworkopen.2023.26352 (PMC10391303; doi:10.1001/jamanetworkopen.2023.26352)
Supplement: Supplement 1. — eTable 1. Coding Information eTable 2. Hospital Factors Associated With Unhoused Pregnancy eTable 3. Sensitivity Analysis for Severe Maternal Morbidity eTable 4. Contraceptive and Sterilization Choices Among Unhoused Pregnant Patients [file jamanetwopen-e2326352-s001.pdf]

Supplemental Online Content

Green JM, Fabricant SP, Duval CJ, et al. Trends, characteristics, and maternal morbidity associated with unhoused status in pregnancy. *JAMA Netw Open*. 2023;6(7):e2326352. doi:10.1001/jamanetworkopen.2023.26352

eTable 1. Coding Information

eTable 2. Hospital Factors Associated With Unhoused Pregnancy

eTable 3. Sensitivity Analysis for Severe Maternal Morbidity

eTable 4. Contraceptive and Sterilization Choices Among Unhoused Pregnant Patients

This supplemental material has been provided by the authors to give readers additional information about their work.

© 2023 Green JM et al. *JAMA Network Open.*

eTable 1. Coding information.

Characteristic	DRG Codes	ICD-10-CM Codes	ICD-10 PCS-Codes
Unhoused status		Z590[§]	
Vaginal delivery	767, 768, 774, 775, 796, 797, 798, 805, 806, 807	O80	10D07Z3, 10D07Z4, 10D07Z5, 10D07Z6, 10D07Z7, 10D07Z8
Cesarean delivery	765, 766, 783, 784, 785, 786, 787, 788	O82, O7582	10D00Z0, 10D00Z1, 10D00Z2
Obesity		E660, E661, E662, E668, E669, Z683, Z684, O9921	
Asthma		J45	
Hypertensive disorder		O10, O11, O13, O14, O15, O16	
Diabetes mellitus		O24	
Tobacco use		Z720, O9933, F17, T652	
Alcohol use		F10, O9931, T51, Q860	
Illicit drug use		F11, F12, F13, F14, F15, F16, F18, F190, O9932	
Schizophrenia disorder		F20, F25	
Bipolar disorder		F31, F30	
Depressive disorder		F33, F32	
Anxiety disorder		F41	
Adjustment disorder		F432	
Suicidal ideation / attempt		R45851, Z915	
Gonorrhea		O982	
Syphilis		O981	
Hepatitis virus		O984	
Herpes		A60	
Tuberculosis		A15, A17, A18, A19	
Prior uterine scar		O342	
Uterine myoma		D25, O341	
Uterine anomaly		Q51, O340	
Grand multiparity		O094, Z641	
Prior pregnancy losses		O262	
Excess gaining weight		O260	
Gestational age		Z3A	
Placenta previa		O44	
Placenta abruption		O45	
Placenta accreta spectrum		O432	
Uterine rupture		O71	
Fetal growth restriction		O365, Z364	
Large for gestational age		O366	
Multifetal gestation		O30, O31, O632, O661, Z372, Z373, Z374, Z375, Z376, Z377, O4302	
Fetal breech position		O321, O641	
Umbilical cord prolapse		O690	
Fetal anomaly		O35	
Fetal demise		O364	

© 2023 Green JM et al. *JAMA Network Open.*

Polyhydramnios		O40	
Oligohydramnios		O410	
**Characteristic**	**DRG Codes**	**ICD-10-CM Codes**	**ICD-10 PCS-Codes**
PROM		O42	
Chorioamnionitis		O411	
Subdermal implant		Z30017	
Intrauterine device		Z30430, Z30014	0UH90HZ, 0UH97HZ, 0UH98HZ, 0UHC7HZ, 0UHC8HZ
Surgical sterilization			0U57, 0UL7, 0UF7, 0UB7, 0UT7
Sever maternal morbidity		**	**

§ Described as "Persons lacking permanent or reliable shelter, variously due to poverty, lack of affordable housing, mental illness, substance abuse, juvenile alienation, or other factors."
** Per Centers for Disease and Control Prevention definition (https://www.cdc.gov/reproductivehealth/maternalinfanthealth/smm/severe-morbidity-ICD.htm)
* Excluded cases with cesarean or vaginal delivery codes.
Abbreviations: DRG, Diagnosis-Related Group; ICD, International Classification of Disease; CM, Clinical Modification; PCS, Procedure Coding System.

© 2023 Green JM et al. *JAMA Network Open.*

eTable 2. Hospital characteristics associated with unhoused status.

Characteristic	No. (%) [a]	Prevalence of unhoused status[b]	aOR (95%CI) [c]
No.	18076440 (100)	104.9	
**Hospital region**			
Northeast	2883759 (16.0)	113.2	1 (reference)
Midwest	3805408 (21.1)	94.2	0.72 (0.69-0.76)
South	7103390 (39.3)	62.4	0.50 (0.48-0.52)
West	4283883 (23.7)	179.4	1.77 (1.69-1.85)
**Hospital bed capacity**			
Small	3484963 (19.3)	86.1	0.79 (0.75-0.82)
Mid	5455064 (30.2)	83.0	0.85 (0.82-0.88)
Large	9136413 (50.5)	125.3	1 (reference)
**Hospital location / teaching**			
Rural	1636472 (9.1)	46.4	0.29 (0.27-0.31)
Urban non-teaching	3653763 (20.2)	56.2	0.55 (0.53-0.58)
Urban teaching	12786205 (70.7)	126.3	1 (reference)

[a] Number with percentage per column.

[b] Row percentage per 100,000 patients.

[c] A binary logistic regression model for multivariable analysis. All the covariates in Tables 2-3 and eTable 2 were entered in the model (all were statistically significant in univariable analysis). Results for patient and pregnancy characteristics are shown in Tables 2 and 3, respectively.

Abbreviations: aOR, adjusted-odds ratio; and CI, confidence interval.

© 2023 Green JM et al. *JAMA Network Open.*

eTable 3. Sensitivity analysis for severe maternal morbidity.

	Outcome rate [a]		Adjusted model [b]
Characteristic	Housed status	Unhoused Status	aOR (95%CI) [c]
**Age (y)**			
<25	18.8	45.6	2.01 (1.76-2.28)
25-29	15.6	43.4	2.02 (1.77-2.30)
30-34	16.1	55.1	2.47 (2.16-2.82)
≥35	21.8	83.9	2.79 (2.46-3.18)
**Race/ethnicity**			
Asian	18.8	96.4	4.07 (2.92-5.69)
Black	27.5	55.3	1.70 (1.52-1.90)
Hispanic	19.9	55.4	2.32 (1.98-2.72)
Native American	25.9	52.1	1.77 (1.18-2.65)
White	13.7	48.2	2.75 (2.46-3.06)
**Registry**			
Northeast	21.0	36.8	1.22 (1.01-1.46)
Midwest	14.3	41.8	2.02 (1.71-2.39)
South	18.4	54.1	2.09 (1.83-2.39)
West	17.1	66.4	3.14 (2.86-3.44)
**Other factor**			
Mental health condition	25.7	46.3	1.42 (1.27-1.59)
Substance use	24.2	60.3	2.17 (2.00-2.35)
Mental health / substance	24.4	55.9	1.92 (1.78-2.07)

[a] Outcome rates for any measured severe maternal morbidity per 1,000 hospital deliveries.

[b] Modeling followed the main cohort (Table 4).

[c] Effect size for unhoused patients compared to housed patients on outcome measures (any severe maternal morbidity). The housed group served as the referent.

Abbreviations: aOR, adjusted odds ratio; CI, confidence interval.

© 2023 Green JM et al. *JAMA Network Open.*

eTable 4. Contraceptive and sterilization choices among unhoused pregnant patients.

Parameters	Outcome rates [a]		Adjusted model [b]
	Housed status	Unhoused status	aOR (95%CI) [c]
Subdermal contraceptive implant	3.5	32.1	6.91 (6.31-7.55)
Intrauterine device	5.6	30.9	4.20 (3.84-4.60)
Surgical sterilization	61.0	61.8	1.01 (0.95-1.08)

[a] Outcome rates per 1,000 hospital deliveries among patients who did not have hysterectomy nor death event (2017-2020 due to availability for subdermal contraceptive implant information).

[b] Adjusted for propensity score followed the main cohort, and grand multiparity, gestational age at delivery, multifetal gestation, and intrauterine fetal demise.

[c] Effect size for unhoused patients compared to housed patients on outcome measures. The housed group served as the referent.

Abbreviations: aOR, adjusted-odds ratio; and CI, confidence interval.

© 2023 Green JM et al. *JAMA Network Open*
