# Supplementary material for: Trends, Characteristics, and Maternal Morbidity Associated With Unhoused Status in Pregnancy
Source: JAMA Netw Open. 2023 Jul 31;6(7):e2326352. doi: 10.1001/jamanetworkopen.2023.26352 (PMC10391303; doi:10.1001/jamanetworkopen.2023.26352)
Supplement: Supplement 2. — Data Sharing Statement [file jamanetwopen-e2326352-s002.pdf]

Data Sharing Statement

Green. Trends, Characteristics, and Maternal Morbidity Associated With Unhoused Status in Pregnancy. *JAMA Netw Open*. Published July 31, 2023.
doi:10.1001/jamanetworkopen.2023.26352

Data
Data available: No

Additional Information
Explanation for why data not available: Data sharing statement: The data on which this study is based are publicly available upon request at Healthcare Cost and Utilization Project, Agency for Healthcare Research and Quality. https://www.hcup-us.ahrq.gov/nisoverview.jsp
